# Dy-Modified Mn/TiO_2_ Catalyst Used for the Selective Catalytic Reduction of NO in Ammonia at Low Temperatures

**DOI:** 10.3390/molecules29010277

**Published:** 2024-01-04

**Authors:** Bing Xu, Zhen Wang, Jie Hu, Lei Zhang, Zhipeng Zhang, Hongtan Liang, Yong Zhang, Guozhi Fan

**Affiliations:** Hubei Provincial Engineering Technology Research Center of Agricultural and Sideline Resources, Chemical Engineering and Utilization, School of Chemistry and Environmental Engineering, Wuhan Polytechnic University, Wuhan 430023, China; wz45151204@163.com (Z.W.); hujie9231@126.com (J.H.); zhanglei@whpu.edu.cn (L.Z.); m17663252107@163.com (Z.Z.); 18883632995@163.com (H.L.); yongzhang@whpu.edu.cn (Y.Z.); fgzcch@whpu.edu.cn (G.F.)

**Keywords:** NH_3_-SCR de-NO_x_, Dy, Mn/TiO_2_ catalyst, low-temperature catalysis

## Abstract

A novel Mn/TiO_2_ catalyst, prepared through modification with the rare-earth metal Dy, has been employed for low-temperature selective catalytic reduction (SCR) denitrification. Anatase TiO_2_, with its large specific surface area, serves as the carrier. The active component MnO_x_ on the TiO_2_ carrier is modified using Dy. Dy_x_Mn/TiO_2_, prepared via the impregnation method, exhibited remarkable catalytic performance in the SCR of NO with NH_3_ as the reducing agent at low temperatures. Experiments and characterization revealed that the introduction of a suitable amount of the rare-earth metal Dy can effectively enhance the catalyst’s specific surface area and the gas–solid contact area in catalytic reactions. It also significantly increases the concentration of Mn^4+^, chemisorbed oxygen, and weak acid sites on the catalyst surface. This leads to a notable improvement in the reduction performance of the DyMn/TiO_2_ catalyst, ultimately contributing to the improvement of the NH_3_-SCR denitrification performance at low temperatures. At 100 °C and a space velocity of 24,000 h^−1^, the Dy_0.1_Mn/TiO_2_ catalyst can achieve a 98% conversion rate of NO_x_. Furthermore, its active temperature point decreases by 60 °C after the modification, highlighting exceptional catalytic efficacy at low temperatures. By doubling the space velocity, the NO_x_ conversion rate of the catalyst can still reach 96% at 130 °C, indicating significant operational flexibility. The selectivity of N_2_ remained stable at over 95% before reaching 240 °C.

## 1. Introduction

Due to the swift advancement of the industrial economy, the issue of environmental pollution has become progressively more pronounced and has gradually garnered widespread concern within society [1,2,3]. Nitrogen oxides (primarily NO), as a crucial air pollutant, significantly affect the chemical composition of the troposphere and are responsible for producing well-known phenomena such as the greenhouse effect, acid rain, PM2.5, and photochemical smog [4,5,6,7,8,9]. As a result, the emission of nitrogen oxides has garnered widespread concern. Countries worldwide have successively established stringent emission standards for nitrogen oxides in response to air pollution. Strict rules and regulations promote the rapid development of technology for treating NO_x_ [10,11,12,13,14,15].

Currently, there are three primary methods of controlling nitrogen oxides, which include raw material control technology prior to combustion, combustion process control technology, and post-combustion control technology. Currently, the initial two techniques can effectively decrease the overall volume of nitrogen oxides, yet meeting the rigorous emission norms proves to be a challenge. The post-combustion control technology has emerged as the cornerstone for complying with emission standards [16,17,18,19,20]. Currently, the most feasible methods for reducing NO_x_ emissions primarily involve selective non-catalytic reduction (SNCR) and selective catalytic reduction (SCR). Of these, selective catalytic reduction of NO_x_ using ammonia as the reductant (NH_3_-SCR) is the most widely used and effective technology in the flue gas denitrification industry. Currently, the V_2_O_5_-WO_3_/TiO_2_ catalyst, the most frequently employed in NH_3_-SCR technology, exhibits outstanding denitrification performance within the temperature range of 280 °C to 400 °C. However, V_2_O_5_-WO_3_/TiO_2_ catalysts have activity at high temperatures and a narrow temperature window. According to the characteristics of high flue gas and sulfur content, the catalyst is prone to poisoning failure and high denitration cost [21,22,23,24,25,26]. The V_2_O_5_ contained in the spent catalyst after use exhibits biological toxicity, posing a threat to both the ecological environment and human health. Regulations have been issued by various countries globally to dispose of invalid V_2_O_5_-WO_3_/TiO_2_ catalysts as hazardous wastes, resulting in increased disposal costs. Therefore, the development of new low-temperature and harmless catalysts provides the NH_3_-SCR process with advantages such as low energy consumption, low investment cost, and long catalyst life. Simultaneously, it is imperative that the non-toxic nature of the failed catalyst toward the environment is emphasized [27,28,29,30,31,32,33,34,35,36,37].

Zhang et al. [17] successfully prepared Cu-SAPO-34 and Mn/SAPO-34 catalysts through hydrothermal synthesis. Through the study of the low-temperature NH_3_-SCR denitrification activity of the two catalysts, it was discovered that Mn had a more significant role in enhancing the catalyst’s activity at low temperatures. The analysis of characterization revealed that MnO_2_ exhibits a remarkable catalytic function in the oxidation of NO, thereby significantly enhancing the rapid progress of the SCR reaction.

In their study, Qi et al. [31] examined the SCR reaction mechanism of the MnO_x_-CeO_2_ catalyst system, which contains rare-earth elements, at low temperatures. The findings revealed that NH_3_, when adsorbed on the Lewis acid sites on the catalyst surface, reacts with gaseous NO upon activation, resulting in the conversion of all intermediate products to NH_2_NO, which subsequently decomposes to produce N_2_ and water. Among them, Ce possesses a specific alkalinity, which facilitates the interaction of oxygen ions in the surrounding environment with NO_x_, thereby oxidizing NO_x_.

Niu et al. [38] were the first to modify Mn/TiO_2_ with Tm, leading to a significant enhancement of the catalyst’s selective catalytic activity at low temperatures. Upon addition of an appropriate amount of Tm, it was discovered that the concentration of Mn^4+^ on the catalyst’s surface, the chemisorption of oxygen, NO, and NH_3_, as well as the reduction performance of the Tm-Mn/TiO_2_ catalyst, were significantly improved. This enhancement is beneficial for improving the NH_3_-SCR reaction’s effectiveness on NO_x_.

To enhance the study of NO_x_ conversion activity at low temperatures, identify additional elements that can modify the catalyst, boost the selectivity for industrial application, and reduce the cost of implementation, our group has conducted extensive research on the modification of the conventional V_2_O_5_-WO_3_/TiO_2_ catalyst system, including the modification of transition metals such as copper, cadmium, and iron. The findings revealed that the reactive temperatures for the catalytic reactions were all above 240 °C, which is unsatisfactory for the current industrial optimization objectives. Therefore, we continue to choose the Mn/TiO_2_ catalytic system for doping modification and utilize Dy from the rare-earth elements as the modifying element to enhance the conversion rate of NO at low temperatures. The results indicate that the inclusion of an adequate quantity of Dy can considerably enhance the conversion activity of Mn/TiO_2_ to NO via NH_3_-SCR technology, specifically at low temperatures ranging from 100 °C to 180 °C.

Rare-earth metals are primarily used as raw materials for rare-earth permanent magnet materials, making them one of the most significant applications of these metals. Rare-earth metals are extensively utilized in new energy batteries. In recent years, the field of denitration catalysts has gradually incorporated the rare-earth metals Tm and Ce, resulting in impressive research outcomes. In our research group, Dy was tasked with modifying the Mn/TiO_2_ catalytic system. The prepared DyMn/TiO_2_ catalyst exhibited outstanding catalytic performance at space velocities of 24,000 and 48,000 h^−1^ below 180 °C; this will greatly reduce the operating cost of SCR denitration. By successfully modifying the MnO_x_/TiO_2_ catalyst with dysprosium, the application scope of rare-earth resources can be expanded. Additionally, these findings will serve as a valuable guide and reference for the widespread adoption of low-temperature catalysts in industrial SCR denitrification processes.

## 2. Results and Discussion

### 2.1. SCR Performance

**Figure 1:**
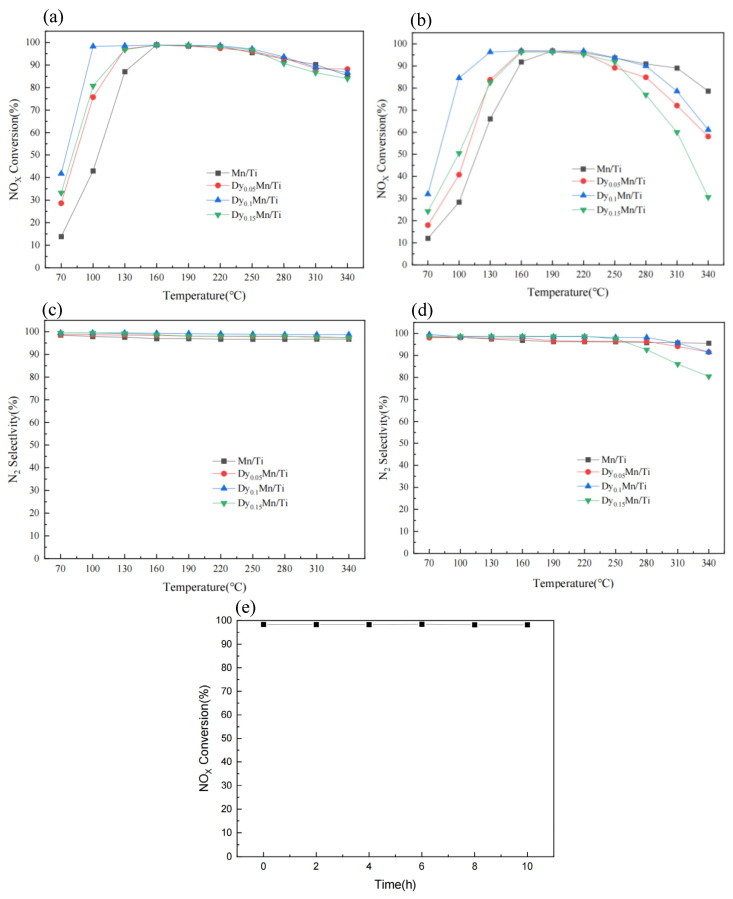
Effect of Dy/Mn molar ratio on NO_x_ conversion (**a**,**b**) and N_2_ selectivity at different space velocities (24,000 h^−1^, 48,000 h^−1^) (**c**,**d**); results of Dy_0.1_Mn/TiO_2_ catalyst stability testing (**e**).

Figure 1 shows the SCR performance test curve of the catalyst sample. At a space velocity of 24,000 h^−1^, Figure 1a illustrates the variation of NO_x_ conversion for Dy_x_Mn/TiO_2_ catalysts with varying molar ratios. Figure 1a reveals that all catalysts exhibit a tendency to initially rise and then decline within the temperature range of 70–340 °C. At 160 °C, the Mn/Ti catalyst achieved a 98% conversion of NO_x_. After the introduction of the rare-earth metal Dy, the NO_x_ conversion of the Dy_x_Mn/TiO_2_ catalyst improved significantly below 160 °C, demonstrating outstanding low-temperature catalytic activity. The conversion of NO_x_ for the Dy_0.05_Mn/TiO_2_ and Dy_0.15_Mn/TiO_2_ catalysts reached 98% at 130 °C, while the Dy_0.1_Mn/TiO_2_ catalyst even achieved a 98% NO_x_ conversion at 100 °C. Additionally, the activity temperature point decreased by 60 °C compared to its pre-modification state, demonstrating outstanding catalytic performance at low temperatures. At a space velocity of 48,000 h^−1^, Figure 1b illustrates the variation of NO_x_ conversion for Dy_x_Mn/TiO_2_ catalysts with varying molar ratios. Upon examination of Figure 1b, it is apparent that the catalytic capacity of all catalysts has diminished. However, the NO_x_ conversion efficiency of the Dy_0.1_Mn/TiO_2_ catalyst surpasses that of other catalysts, attaining a remarkable 96% at 130 °C. The results indicate that the introduction of the rare-earth metal Dy can significantly enhance the NO_x_ conversion of the Mn/Ti catalyst, and when the molar ratio of Dy/Ti is 0.1, the catalyst exhibits outstanding denitration performance.

Simultaneously, the series of prepared catalysts demonstrate notable catalytic activity within the Space velocity range of 24,000 h^−1^ to 48,000 h^−1^, indicating their broad operational flexibility in catalytic reactions and making them highly suitable for industrial application. Figure 1c,d display the N_2_ selectivity of Dy_x_Mn/TiO_2_ catalyst samples when the space velocity is 24,000 h^−1^ and 48,000 h^−1^, respectively, ensuring an N_2_ selectivity of over 95% below 240 °C. At elevated space velocities, when the temperature exceeds 260 °C, the selectivity decreases due to an increased likelihood of side reactions and a reduced contact time and chance between gas and catalyst, primarily attributed to the excessive space velocity. In summary, when the molar ratio of Dy/Ti is 0.1, the Dy_x_Mn/TiO_2_ catalyst sample exhibits the best low-temperature SCR denitrification performance and outstanding N_2_ selectivity. Lastly, the stability of the Dy_0.1_Mn/TiO_2_ catalyst sample was tested at 150 °C. As shown in Figure 1e, the catalytic efficiency of the Dy_0.1_Mn/TiO_2_ catalyst sample consistently remained above 98% throughout the entire test period, demonstrating the catalyst’s exceptional stability.

### 2.2. SEM, Crystallinity, and Porous Property Analyses

**Figure 2:**
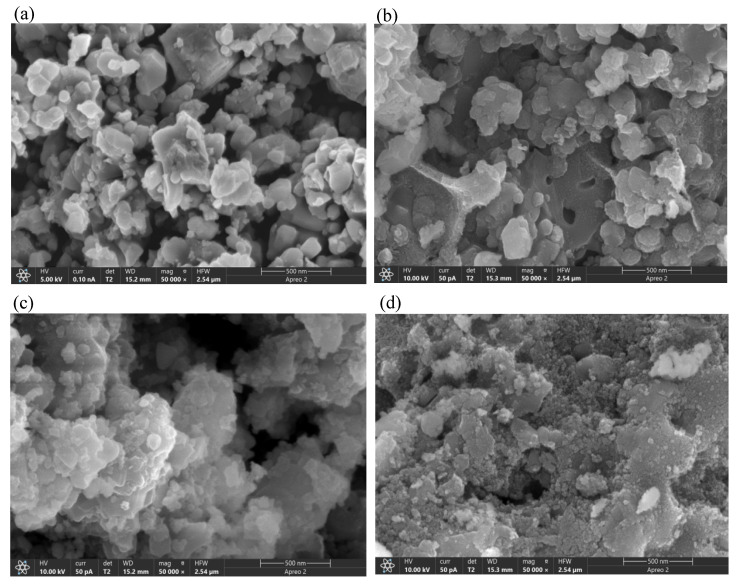
SEM microstructures of the different catalysts. (**a**) Surface morphology of Mn/TiO_2_. (**b**) Surface morphology of Dy_0.5_Mn/TiO_2_. (**c**) Surface morphology of Dy_0.1_Mn/TiO_2_. (**d**) Surface morphology of Dy_0.15_Mn/TiO_2_.

Figure 2 displays the SEM images of the Mn/TiO_2_ catalyst and the Dy_x_Mn/TiO_2_ catalyst. As the molar ratio of Dy increases, the active components on the catalyst surface gradually increase, as evident from Figure 2. However, the surface of the Dy_0.15_Mn/TiO_2_ catalyst exhibited noticeable agglomeration, leading to a decrease in the catalyst’s specific surface area. Concurrently, this phenomenon obscured the active sites on the surface to some extent, ultimately diminishing the catalyst’s SCR catalytic activity. As shown in Figure 2c, the Dy_0.1_Mn/TiO_2_ catalyst boasts a considerable amount of active components uniformly distributed across its surface, thereby manifesting commendable catalytic activity in SCR catalytic performance experiments.

**Figure 3:**
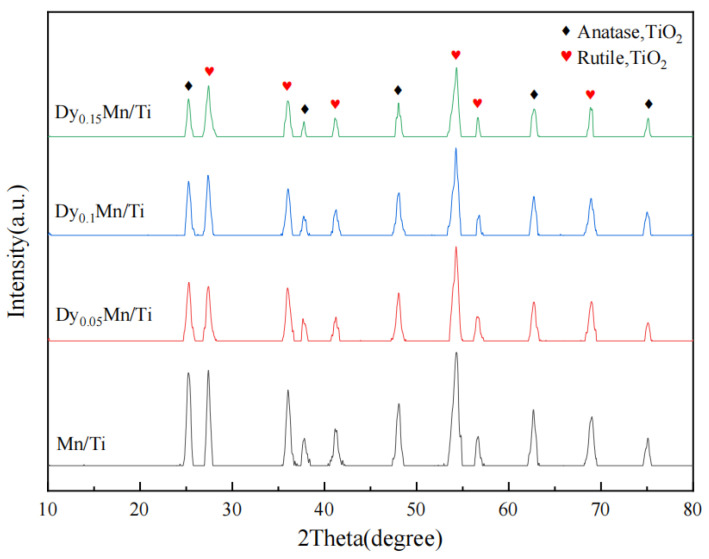
XRD patterns of the different catalysts.

Figure 3 displays the XRD patterns of the Dy_x_Mn/TiO_2_ catalyst. All catalysts exhibited comparable XRD diffraction peaks, suggesting that the addition of the rare-earth metal Dy did not alter the crystal structure of the catalyst. Characteristic peaks of anatase TiO_2_ were observed at 25.8°, 38.0°, 47.6°, 54.5°, 62.8°, 69.5°, and 75.2°. Anatase TiO_2_ is more beneficial for enhancing the catalytic performance of SCR [35]. As shown in Figure 3, no characteristic peaks of Mn and Dy oxides were observed on the surface of the catalyst sample. This suggests that the oxides of Mn and Dy are either highly dispersed on the surface of the catalyst or exist in an amorphous form, which cannot be detected by XRD [39]. Upon the introduction of the rare-earth metal Dy, as the Dy/Ti molar ratio increases, the diffraction peak intensity exhibits a trend of initial decrease, subsequent increase, and subsequent decrease. The introduction of Dy into the system indicates a reduction in the crystallinity of the TiO_2_ support. Table 1 presents the ICP–OES analysis of catalysts. The results indicate that the actual loading mass of Dy in each catalyst essentially aligns with the theoretical value. It also demonstrates that the prepared catalyst does indeed contain a specific amount of Mn and Dy oxides, which to some extent corroborates the XRD data analysis.

**Figure 4:**
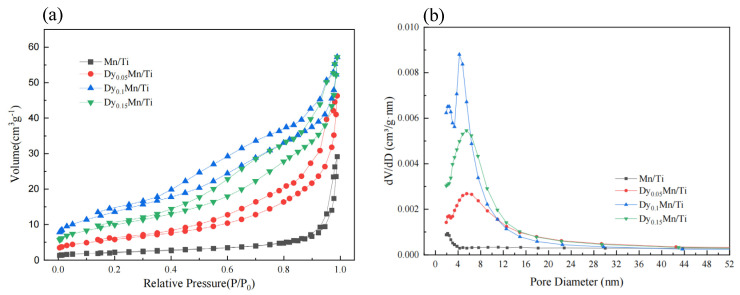
(**a**) Nitrogen adsorption-esorption curve of the different catalysts. (**b**) Pore size distribution curve of the different catalysts.

Figure 4 depicts the adsorption–desorption isotherms of all catalysts. The catalysts exhibit type IV isotherms and type H2 hysteresis loops, which are hallmarks of mesoporous materials. The measurement and calculation of specific surface area, pore volume, and pore diameter are based on BET and BJH equations, as detailed in Table 2 below. The specific surface area of the Mn/TiO_2_ catalyst is only 7.94 m^2^/g. With the introduction of Dy, the specific surface area initially increases and then decreases. The pore volume becomes larger but the pore size becomes smaller. The specific surface area of the Dy_0.1_Mn/TiO_2_ catalyst is as high as 49.47 m^2^/g, which is higher than 37.01 m^2^/g in the literature [39]. The large surface area of the catalyst facilitates the rapid adsorption of reactants and the desorption of products on the catalyst surface, thereby enhancing the low-temperature SCR catalytic performance of the catalyst [38]. The results indicate that the specific surface area of the catalyst and the gas–solid contact area of the catalytic reaction can be significantly increased when an appropriate amount of the rare-earth metal Dy is introduced, which also enhances the SCR denitrification activity of the catalyst.

### 2.3. XPS Analysis

**Figure 5:**
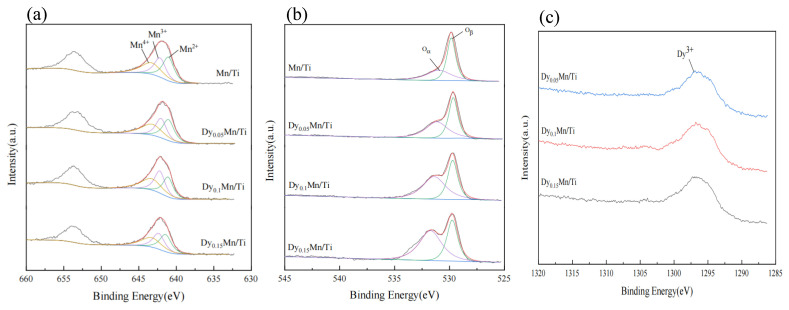
XPS of the different catalysts: (**a**) Mn 2p, (**b**) O 1s, (**c**) Dy 4d.

Figure 5 displays the X-ray photoelectron spectroscopy (XPS) characterization of the Dy_x_Mn/TiO_2_ catalyst, aiming to investigate the variations in elemental valence on the surface of various catalysts. It involves the separation and fitting of characteristic peaks for Mn, O, and Dy elements in each catalyst, and the computation of the relative concentrations of Mn, O, and Dy elements in varying valence states on the surface of each catalyst. As illustrated in Figure 5a, the photoelectron peaks for Mn 2p3/2 and Mn 2p1/2 are situated around 642.0 eV and 653.5 eV, respectively. Mn 2p3/2 was categorized into three electronic peaks, namely Mn^2+^ (641.2 ± 0.1 eV), Mn^3+^ (642.3 ± 0.1 eV), and Mn^4+^ (643.8 ± 0.1 eV) [38]. According to relevant literature [39], high levels of Mn^4+^ promote the NH_3_-SCR catalytic reduction reaction. Table 2 shows that with the addition of the rare-earth metal Dy, the Mn^4+^/(Mn^2+^ + Mn^3+^ + Mn^4+^) ratio first increases and then decreases. The Dy_0.1_Mn/TiO_2_ catalyst exhibits the highest ratio, aligning with the results of the SCR performance test. This aligns with the literature’s conclusion: Mn^4+^ concentration is a crucial factor in determining the catalyst’s low-temperature catalytic activity.

As shown in Figure 5b, all catalysts exhibit two overlapping peaks around 529.8 eV and 531.5 eV [38]. The peak at 529.8 eV belongs to lattice oxygen (denoted as O_β_), while the peak at 531.5 eV belongs to adsorbed oxygen (denoted as O_α_). According to a literature report [39], O_α_ has a higher mobility than O_β_. It can be converted to lattice oxygen at oxygen vacancies on the catalyst surface, thereby enhancing the oxidation ability of NO at low temperatures and promoting the rapid progress of the SCR reaction. Table 2 reveals that with the addition of the rare-earth metal Dy, the ratio of O_α_/(O_α_ + O_β_) on the catalyst surface gradually increases. This suggests that the amount of adsorbed oxygen is a pivotal factor influencing the catalyst’s low-temperature catalytic performance. The location of Dy 4d is indicated to be around 1294 eV, as depicted in Figure 5c, which corresponds to the Dy^3+^ on the catalyst surface.

### 2.4. H_2_-TPR, NH_3_-TPD Analysis

**Figure 6:**
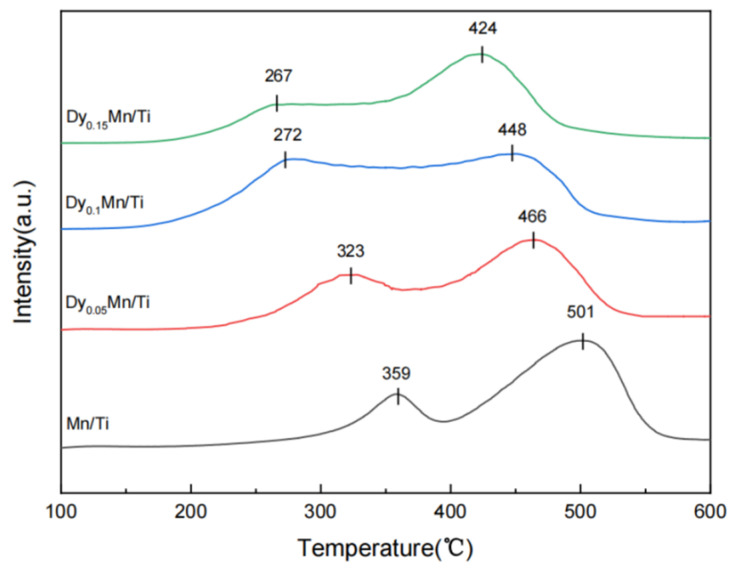
H_2_-TPR analysis of the different catalysts.

The H_2_-TPR experiment is conducted to investigate the redox behavior of the Dy_x_Mn/TiO_2_ catalyst. The redox properties of catalysts significantly determine their catalytic properties. As illustrated in Figure 6, all catalysts exhibit two broad, overlapping reduction peaks within the temperature range of 150–550 °C. For the Mn/Ti catalyst, the reduction peak around 359 °C is attributed to the reduction process of MnO_2_ → Mn_2_O_3_ [31,38]. The reduction peak within the temperature range of 420–520 °C is attributed to the reduction process of Mn_2_O_3_ → Mn_3_O_4_ → MnO [39]. Compared to the peak of Mn/TiO_2_, the reduction peak of the Dy–Mn/Ti catalyst shifted towards lower temperatures due to the synergistic effect between Dy and Mn/TiO_2_. Among them, the Dy_0.1_Mn/TiO_2_ catalyst and the Dy_0.15_Mn/TiO_2_ catalyst exhibit the lowest reduction peak temperatures. The peak performance of the Dy_0.1_Mn/TiO_2_ catalyst is notably broader and encompasses regions of lower temperature. Upon calculating the peak area, it is observed that the Dy_0.1_Mn/TiO_2_ catalyst boasts the largest peak area, as illustrated in Table 3. The H_2_-TPR results indicate that the Dy_0.1_Mn/TiO_2_ catalyst exhibits the optimal reduction behavior at low temperatures and demonstrates the highest catalytic activity among the SCR catalysts, corroborating the findings from the prior SCR catalytic performance tests.

**Figure 7:**
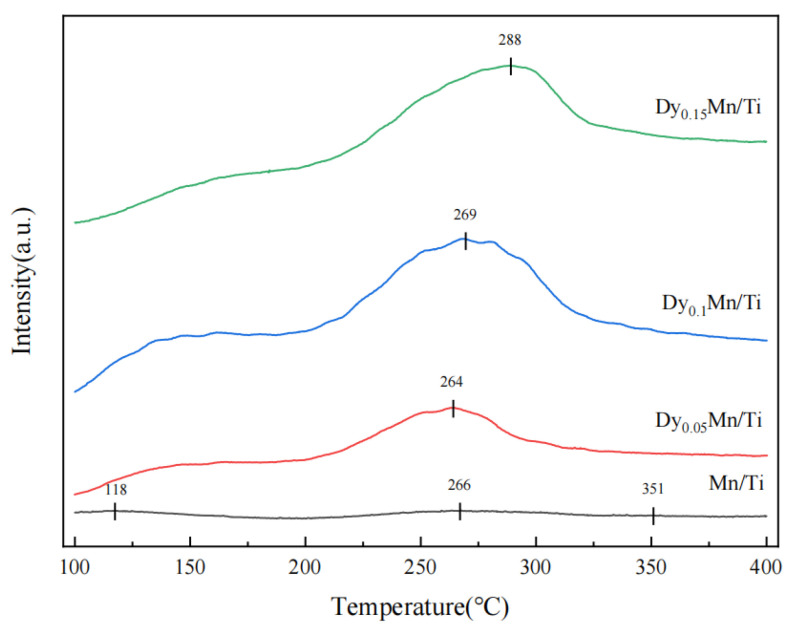
NH_3_-TPD analysis of the different catalysts.

In the NH_3_-SCR reaction, the adsorption capacity of the catalyst for NH_3_ is crucial. Figure 7 shows the NH_3_-TPD characterization results for all Dy_x_Mn/TiO_2_ catalysts. Three weak desorption peaks can be observed for the Mn/TiO_2_ catalyst. The peak around 118 °C is attributed to the physical adsorption of NH_3_ on the catalyst, the peak around 266 °C is attributed to the desorption peak of NH_3_ adsorbed on the Bronsted acid site (weak acid), and the peak around 351 °C is attributed to the desorption peak of NH_3_ adsorbed on the Lewis acid site (strong acid) [38]. Upon introduction of the rare-earth metal Dy, the desorption peak on weak acid significantly intensified. As the Dy content increased, the area of the desorption peak initially expanded and then diminished. As illustrated in Table 3, it is observed that the peak area of NH_3_ desorption for the Dy_0.1_Mn/TiO_2_ catalyst is the largest among all catalysts, suggesting the highest number of weak acid sites on the catalyst surface. According to literature reports [38], weak acids are crucial to the low-temperature catalytic activity of catalysts. Consequently, the moderate incorporation of Dy notably enhances the adsorption capacity of NH_3_ on the catalyst surface, substantially increases the number of weak acid sites on the catalyst surface, and consequently boosts the catalyst’s activity at low temperatures. The results of the NH_3_-TPD characterization experiments further corroborate and validate the previous results of the NH_3_-SCR performance tests.

## 3. Experiment

### 3.1. Synthesis Catalyst

**Figure 8:**
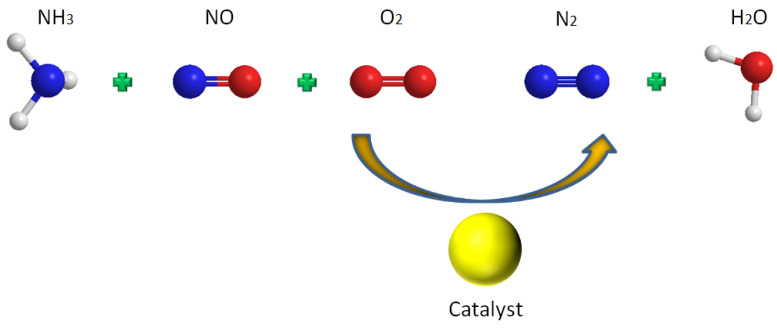
Main schematic diagram of NH_3_-SCR denitration.

A series of DyMn/TiO_2_ catalysts with varying Dy/Mn molar ratios were prepared using the impregnation method. The detailed synthesis procedure is as follows: Weigh an appropriate amount of Dysprosium Nitrate (AR, purchased from Shanghai Mcleam Biochemical Technology Co., Ltd., Shanghai, China) and manganese acetate (AR, purchased from Alddin Reagent Co., Ltd., Shanghai, China) into a beaker that holds 50 mL of deionized water. To facilitate dissolution, place the beaker into a magnetic water bath stirring pot and stir thoroughly. Weigh an appropriate amount of titanium dioxide powder (AR, purchased from Sinopharm Chemical Reagent Co., Ltd., Shanghai, China) and add it gradually to the beaker. Stir at room temperature for 2 h, and then raise the temperature to 90 °C using the water bath. Continue stirring until the water evaporates to a viscous state. Then, remove the beaker and dry it in a 110 °C drying oven for 15 h to obtain a dry, block-like solid. Subsequently, transfer the block-shaped solid to a quartz crucible and roast it in a muffle furnace at 400 °C (with a heating rate of 6 °C/min) for four hours in an air atmosphere to yield the calcined solid particles. Finally, make a catalyst powder with a particle diameter ranging from 0.25 to 0.45 mm through tablet pressing, grinding, and sieving to achieve a mesh size of 40–60. The synthesized catalyst series is denoted by the symbol Dy_x_Mn/TiO_2_, where x represents the molar ratio of Dy to Ti (Mn:Ti = 0.3:1; the mass fraction of Dy was 7.02%, 13%, and 18.13%, respectively). Additionally, when x is 0, it corresponds to the Mn/TiO_2_ catalyst, also prepared via the impregnation method.The reaction mechanism is shown in Figure 8.

### 3.2. Characterization of Catalysts

The phase composition and crystal phase composition of the catalyst were analyzed using an X-ray diffractometer (D8ADVANCE, Bruker Corporation, Bremen, Germany), with cu-k α serving as the radiation source. The tube voltage was set to 40 kV, the tube current to 30 mA, the XRD scanning range to 10–80°, and the step size to 0.02°. A scanning electron microscope (JSM-7610FPlus, JEOL Co., Ltd., Tokyo, Japan) was used to observe the morphology of aerogel under an accelerating voltage of 15 kV. The specific surface area and pore structure of the samples were determined using a specific surface area and pore volume aperture analyzer (ASAP2460, Micromeritics, Norcross, GA, USA). The calculation of test data was performed according to BET analysis. The X-ray photoelectron spectrometer (Escalab 250xi, Thermo Fisher Scientific, Waltham, MA, USA) was employed for the XPS test, with the aim of investigating the valence state of atoms present on the surface of the catalyst. The binding energies of the measured elements were corrected using the C 1s peak (284.6 eV). An analysis was conducted using an Al Kα X-ray with a tube voltage of 1486.6 eV. The content of metal elements in the catalyst was determined by inductively coupled plasma atomic emission spectrometry (ICPOES 730, Aglient Technology Co., Ltd., Santa Clara, CA, USA).

The H_2_-TPR experiment employed the BELCAT-A system (BELCAT-A, Bayer, Tokyo, Japan) for temperature-programmed reduction, aimed at examining the impact of the catalyst’s redox property on its catalytic performance. Test procedure: Weigh 100 mg of sample and transfer it into a reaction tube. Dry it at a rate of 10 °C/min, increasing from room temperature to 300 °C. Use a high-purity nitrogen flow rate (30 mL/min) to purge it for 1 h, and then allow it to cool down to 50 °C. Convert N_2_ to a reducing gas (10% H_2_/N_2_) for purge purposes, remove any residual N_2_ on the sample surface and within the pipeline, maintaining a flow rate of 30 mL/min, then increase the temperature from 50 °C to 700 °C at a rate of 10 °C/min. Record the TCD signal value.

The NH_3_-TPD analysis was also conducted using the BELCAT-A system (BELCAT-A, Bayer, Japan) to investigate the impact of acidity on the catalytic performance of the catalyst. Test procedure: Weigh 50–200 mg of sample and transfer it into a reaction tube. Program the temperature to rise to 300 °C at a rate of 10 °C/min for drying pretreatment. Purge it with a N_2_ flow of 30 mL/min for 1 h and cool it to 50 °C. Subsequently, introduce a 10% NH_3_/N_2_ mixture (30 mL/min) and leave it to saturate for an hour. Afterward, switch on the flow of N_2_ (30 mL/min) to purge for an hour and remove any weakly physically adsorbed NH_3_ on the surface. Finally, under an N_2_ atmosphere, raise the temperature at a rate of 10 °C/minute until it reaches 600 °C for desorption. The desorbed gas is detected using a thermal conductivity detector (TCD).

### 3.3. Characterization of Catalysts

**Figure 9:**
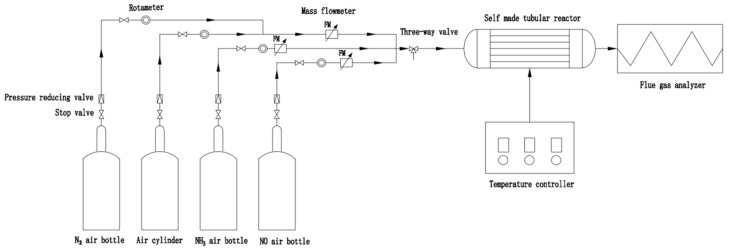
Flow chart of catalyst performance test device.

The laboratory self-made denitration activity evaluation equipment is the experimental device employed for the NH_3_-SCR performance test of the experimental catalyst (Figure 9). The reactor has an inner diameter of 1 cm and a length of 50 cm. The center of the reaction tube is filled with 0.096 g of catalyst, and the temperature of the reactor is monitored using a thermocouple. During the reaction process, the temperature is maintained between 100–400 °C. For every 30 °C increase, the temperature is held for approximately 20 min to stabilize the concentration of each tail gas component. Real-time data are recorded sequentially. Rotor flowmeters and volume flowmeters are utilized to harmoniously regulate the flow of gases from each pathway, stabilizing the overall flow rate of the simulated flue gas atmosphere. Additionally, the gas volume space velocity is adjusted to 24,000 h^−1^ (Flow rate is 300 mL/min) and 48,000 h^−1^, respectively. An online flue gas analyzer (GW-2000) is employed to monitor the NO_x_ content in the reactor’s exhaust, a nitrous oxide detector (FGD2-C-N_2_O) is utilized to measure the N_2_O level in the reaction’s exhaust, and a specific length gas detection tube is used to determine the concentration of ammonia in both the intake air and the exhaust.

(1) $${NO}_{x}conversion\;(\%)=\frac{{\left[{NO}_{x}\right]}_{in}-{\left[{NO}_{x}\right]}_{out}}{{\left[{NO}_{x}\right]}_{in}}\times 100\%$$

(2) $$N_{2}\;selectivity\;(\%)=\frac{{\left[{NO}_{x}\right]}_{in}+{\left[{NH}_{3}\right]}_{in}-{\left[{NO}_{x}\right]}_{out}-{\left[{NH}_{3}\right]}_{out}-{\left[N_{2}O\right]}_{out}}{{\left[{NO}_{x}\right]}_{in}+{\left[{NH}_{3}\right]}_{in}-{\left[{NO}_{x}\right]}_{out}-{\left[{NH}_{3}\right]}_{out}}\times 100\%$$

## 4. Conclusions

An efficient Mn/TiO_2_ catalyst, prepared through modification with the rare-earth metal Dy, has been employed for low-temperature selective catalytic reduction (SCR) denitrification. The Dy_x_Mn/TiO_2_ catalyst, prepared by the impregnation method, exhibited outstanding catalytic performance. Experiments and characterization revealed that the introduction of a suitable amount of the rare-earth metal Dy can effectively enhance the specific surface area of the catalyst and increase the contact area for gas–solid reactions. Concurrently, the incorporation of the rare-earth metal Dy significantly enhances the concentration of Mn^4+^ on the catalyst surface and the chemisorption of oxygen, significantly contributing to the improvement of the catalyst’s activity. Lastly, the SCR catalytic performance was confirmed through NH_3_-TPD and H_2_-TPR experiments. The findings indicate that the incorporation of a suitable amount of the metal Dy significantly enhances the adsorption capacity of NH_3_ on the catalyst surface and effectively augments the number of weak acid sites on the catalyst surface, thereby enhancing the catalyst’s activity at low temperatures, ameliorating the reduction performance of the DyMn/TiO_2_ catalyst, and ultimately bolstering the performance of NH_3_-SCR denitration at low temperatures. The Dy_0.1_Mn/TiO_2_ catalyst can achieve a 98% NO_x_ conversion rate at 100 °C under a space velocity of 24,000 h^−1^. Additionally, its active temperature point is 60 °C lower than before modification, demonstrating an outstanding catalytic performance at low temperatures. After doubling the space velocity, the NO_x_ conversion of the catalyst remains at 96% even at 130 °C, demonstrating substantial operational flexibility. The selectivity to N_2_ remains above 95% up to 240 °C.

## Figures and Tables

**Table 1:** Mass fraction of the Dy_x_Mn/TiO_2_ catalysts measured with ICP–OES.

Catalyst Sample	Theoretical Load Capacity	Actual Load Capacity
Mn/TiO_2_	15.55% Mn	14.41% Mn
Dy_0.05_Mn/TiO_2_	14.29% Mn, 7.03% Dy	13.07% Mn, 6.70% Dy
Dy_0.1_Mn/TiO_2_	13.22% Mn, 13.02% Dy	12.18% Mn, 13.20% Dy
Dy_0.15_Mn/TiO_2_	12.30% Mn 18.18% Dy	13.02% Mn, 18.85% Dy

**Table 2:** The pore analysis data of the different catalysts.

Catalyst Sample	BET Surface Area (m^2^/g)	Pore Volume (cm^3^/g)	Average Pore Diameter (nm)
Mn/TiO_2_	7.94	0.044	26.78
Dy_0.05_Mn/TiO_2_	21.00	0.071	13.62
Dy_0.1_Mn/TiO_2_	49.47	0.086	7.53
Dy_0.15_Mn/TiO_2_	35.91	0.087	9.88

**Table 3:** The valence concentration of elements on the surface of the different catalysts and H_2_ reduction peak area.

Catalyst Sample	Mn^4+^/Mn^n+^(%)	O_α_/(O_α_ + O_β_) (%)	H_2_ Reduction Peak Area (%)	NH_3_-TPD Peak Area (%)
Mn/TiO_2_	31.75	39.59	76.91	7.28
Dy_0.05_Mn/TiO_2_	33.64	49.36	73.29	44.37
Dy_0.1_Mn/TiO_2_	35.06	60.21	100.00	100.00
Dy_0.15_Mn/TiO_2_	33.02	61.32	77.64	78.88

Note: The peak area of H_2_ reduction and NH_3_-TPD are normalized with Dy_0.1_Mn/TiO_2_ as the standard.

## Data Availability

The data presented in this study are available on request from the corresponding author.

## References

[1] Li Y., Leng X., Ma S., Zhang T., Yuan F., Niu X., Zhu Y. (2020). Effects of Mo addition on the NH_3_-SCR of NO reaction over Mo_a_ MnTi_10_O_x_ (a = 0.2, 0.4, 0.6 and 0.8): Synergistic action between redox and acidity. Catal. Today.

[2] Zhou J., Guo R., Zhang X., Liu Y., Pan W. (2021). Cerium oxide—Based catalysts for low-temperature selective catalytic reduction of NO_x_ with NH_3_: A review. Energ. Fuel.

[3] Xie H., He P., Chen C., Yang C., Chai S., Wang N., Ge C. (2023). Improvement of Sb-Modified Mn-Ce/TiO_2_ Catalyst for SO_2_ and H_2_O Resistance at Low-Temperature SCR. Catal. Lett..

[4] Jiang H., Wang H., Kuang L., Li G., Zhang M. (2014). Synthesis of MnO_x_–CeO_2_·NO_x_ catalysts by polyvinyl pyrrolidone–assisted supercritical antisolvent precipitation. J. Mater. Res..

[5] Kapteijn F., Singoredjo L., Andreini A., Moulijn J.A. (1994). Activity and selectivity of pure manganese oxides in the selective catalytic reduction of nitric oxide with ammonia. Appl. Catal. B-Environ..

[6] Dimitrios K.P., Pappas A., Thirupathi B., Punit B., Panagiotis G.S. (2016). Novel manganese oxide confined interweaved titania nanotubes for the low-temperature Selective Catalytic Reduction (SCR) of NO_x_ by NH_3_—ScienceDirect. J. Catal..

[7] Yang S., Qi F., Liao Y., Xiong S., Lan Y., Shan W., Li J. (2014). Dual effect of sulfation on the selective catalytic reduction of NO with NH_3_ over MnO_x_/TiO_2_: Key factor of NH_3_ distribution. Ind. Eng. Chem. Res..

[8] Yang S., Qi F., Xiong S., Dang H., Liao Y., Wong P., Li J. (2016). MnO_x_ supported on Fe-Ti spinel: Anovel Mn based low temperature SCR catalyst with a high N_2_ selectivity. Appl. Catal. B.-Environ..

[9] Zhang B., Zhang S., Liu B. (2019). Comparative study on transition element doped Mn-Zr-Ti-oxides catalysts for the low-temperature selective catalytic reduction of NO with NH_3_. React. Kinet. Mech. Catal..

[10] Zhang B., Liebau M., Suprun W., Liu B., Zhang S., Glaeser R. (2019). Suppression of N_2_O formation by H_2_O and SO_2_ in the selective catalytic reduction of NO with NH_3_ over a Mn/Ti-Si catalyst. Catal. Sci. Technol..

[11] Qiu L., Wang Y., Pang D., Feng O., Zhang C., Cao G. (2016). Characterization and catalytic activity of Mn–Co/TiO_2_ catalysts for NO oxidation to NO_2_ at low temperature. Catalysis.

[12] Qiu L., Meng J., Pang D., Zhang C., Ouyang F. (2015). Reaction and characterization of Co and Ce doped Mn/TiO_2_ catalysts for low-temperature SCR of NO with NH_3_. Cawal. Lett..

[13] Qiu L., Pang D., Zhang C., Meng J., Zhu R., Ouyang F. (2015). In situ IR studies of Co and Ce doped Mn/TiO_2_ catalyst for low-temperature selective catalytic reduction of NO with NH_3_. Appl. Surf. Sci..

[14] Guan B., Zhan R., Lin H., Huang Z. (2014). Review of State of the Art Technologies of Selective Catalytic Reduction of NO_x_ from Diesel Engine Exhaust. Appl. Therm. Eng..

[15] Liu L., Su S., Xu K., Li H., Qing M., Hu S., Wang Y., Xiang J. (2019). Insights into the highly efficient Co modified MnSm/Ti catalyst for selective catalytic reduction of NO with NH_3_ at low temperature. Fuel.

[16] De La Torre U., Pereda-Ayo B., González-Marcos J.A., Gutiérrez-Ortiz M.A., González-Velasco J.R. (2016). Performance of Cu-ZSM-5 in a coupled monolith NSR-SCR system for NO_x_ removal in lean-burn engine exhaust. Top. Catal..

[17] Zhang M., Cao H., Chen Y., Jiang H. (2019). Role of Mn: Promotion of fast-SCR for Cu-SAPO-34 in low-temperature selective catalytic reduction with ammonia. Catal. Surv. Asia.

[18] Liu X., Zhao Z., Ning R., Qin Y., Zhu T., Liu F. (2019). Ce-Doped V_2_O_5_-WO_3_/TiO_2_ with low vanadium loadings as SCR catalysts and the resistance of H_2_O and SO_2_. Catal. Lett..

[19] Gao Y., Jiang W., Luan T., Li H., Zhang W., Feng W., Jiang H. (2019). High-efficiency catalytic conversion of NO_x_ by the synergy of nanocatalyst and plasma: Effect of Mn-based bimetallic active species. Catalysts.

[20] Sun P., Guo R., Liu S., Wang S., Pan W., Li M. (2017). The enhanced performance of MnO_x_ catalyst for NH_3_-SCR reaction by the modification with Eu. Appl. Catal. A-Gen..

[21] Gao F., Tang X., Yi H., Li J., Zhao S., Wang J., Chu C., Li C. (2017). Promotional mechanisms of activity and SO_2_ tolerance of Co- or Ni-doped MnO_x_-CeO_2_ catalysts for SCR of NO_x_ with NH_3_ at low temperature. Chem. Eng. J..

[22] Gao Y., Luan T., Zhang M., Zhang W., Feng W. (2018). Structure-activity relationship study of Mn/Fe ratio effects on Mn-Fe-Ce-O_x/γ_-Al_2_O_3_ nanocatalyst for NO oxidation and fast SCR reaction. Catalysts.

[23] Liu Z., Chen C., Zhao J., Yang L., Sun K., Zeng L., Pan Y., Liu Y., Liu C. (2020). Study on the NO_2_ production pathways and the role of NO_2_ in fast selective catalytic reduction DeNO_x_ at low-temperature over MnO_x_/TiO_2_ catalyst. Chem. Eng. J..

[24] Li W., Zhang C., Li X., Tan P., Zhou A., Fang Q., Chen G. (2018). Ho-modified Mn-Ce/TiO_2_ for low-temperature SCR of NO with NH_3_: Evaluation and characterization. Chin. J. Catal..

[25] Zhang T., Ma S., Chen L., Li R., Leng X., Li Y., Yuan F., Niu X., Zhu Y. (2019). Effect of Cu doping on the SCR activity over the CumCe_0.1−m_TiO_x_ (m = 0.01, 0.02 and 0.03) catalysts. Appl. Catal. A-Gen..

[26] Wang S., Guo R., Pan W., Li M., Sun P., Liu S., Liu S., Sun X., Liu J. (2017). The deactivation mechanism of Pb on the Ce/TiO_2_ catalyst for the selective catalytic reduction of NO_x_ with NH_3_: TPD and DRIFT studies. Phys. Chem. Chem. Phys..

[27] Yu Z., Xu L., Liang Z., Wang Z. (2019). Doping and immobilization of TiO_2_ with element Na and raschig rings. Chem. Phys. Lett..

[28] Fan Z., Shi J., Gao C., Gao G., Wang B., Wang Y., He C., Niu C. (2018). Gd-modified MnO_x_ for the selective catalytic reduction of NO by NH_3_: The promoting effect of Gd on the catalytic performance and sulfuresistance. Chem. Eng. J..

[29] Xu Q., Su R., Cao L., Li Y., Yang C., Luo Y., Street J., Jiao P., Cai L. (2017). Facile preparation of high-performance Fe-doped Ce-Mn/TiO_2_ catalysts for the low-temperature selective catalytic reduction of NO_x_ with NH_3_. RSC Adv..

[30] Li Y., Li Y., Shi Q., Qiu M., Zhan S. (2017). Novel hollow microspheres Mnx Co_3−x_ O_4_ (x = 1, 2) with remarkable performance for low-temperature selective catalytic reduction of NO with NH_3_. J. Sol.-Gel. Sci. Technol..

[31] Qi G., Yang R.T., Chang R. (2004). MnO_x_—CeO_2_ mixed oxides prepared by co-precipitation for selective catalytic reduction of NO with NH_3_ at low temperatures. Appl. Catal. B-Environ..

[32] Tang X., Hao J., Yi H., Li J. (2007). Low Temperature SCR of NO with NH_3_ over AC/C supported manganese-based monolithic catalysts. Catal. Today.

[33] Cheng K., Liu J., Zhang T., Li J., Duan A. (2014). Effect of Ce doping of TiO_2_ support on NH_3_–SCR activity over V_2_O_5_-WO_3_/CeO_2_-TiO_2_ catalyst. J. Environ. Sci..

[34] Jing W., Guo Q., Hou Y., Ma G., Han X., Huang Z. (2014). Catalytic role of vanadium(V) sulfate on activated Carbon for SO_2_ oxidation and NH_3_–SCR of NO at low temperatures. Catal. Commun..

[35] Liu Z., Zhang S., Li J., Zhu J., Ma L. (2014). Novel V_2_O_5_–CeO_2_/TiO_2_ catalyst with low vanadium loading for the selective catalytic reduction of NO_x_ by NH_3_. Appl. Catal. B-Environ..

[36] Yang S., Xiong S., Liao Y., Xiao X., Qi F., Peng Y., Fu Y., Shan W., Li J. (2014). Mechanism of N_2_O Formation during the Low–Temperature Selective Catalytic Reduction of NO with NH_3_ over Mn-Fe Spinel. Environ. Sci. Technol..

[37] Wang J., Yan Z., Liu L., Chen Y., Wang X. (2014). In Situ DRIFTS Investigation on the SCR of NO with NH_3_ over V_2_O_5_ Catalyst Supported by Activated Semi-Coke. Appl. Surf. Sci..

[38] Niu C., Wang B., Xing Y., Su W., He C., Xiao L., Xu Y., Zhao S., Cheng Y., Shi J. (2021). Thulium modifified MnO_x_/TiO_2_ catalyst for the low-temperature selective catalytic reduction of NO with ammonia. J. Clean. Prod..

[39] Zhang Y., Wu P., Li G., Zhuang K., Shen K., Wang S., Huang T. (2020). Improved activity of Ho-modified Mn/Ti catalysts for the selective catalytic reduction of NO with NH_3_. Environ. Sci. Pollut. Res..

